# Bacterial pathogens occurring in poultry litter#^⁎^

**DOI:** 10.1016/j.psj.2026.108046

**Published:** 2026-10-27

**Authors:** Steven C. Ricke, Ashton E. Rogers, Elena G. Olson, Brett M. Hale

**Affiliations:** a Department of Animal and Dairy Sciences, University of Wisconsin, Madison, WI, 53706, USA; b AgriGro, Inc, Doniphan, MO, 63935, USA

**Keywords:** Poultry litter, Bacterial pathogens, Litter management, Survival, Transmission

## Abstract

As demand for poultry meat continues to increase, commercial broiler production relies on increasingly large-scale facilities and higher stocking densities. In production environments, which prioritize maximum efficiency, environmental management is often overlooked and/or underprioritized. There is an increased risk of bacterial pathogen transmission, environmental contamination, and the accumulation of organic waste, allowing widespread pathogen proliferation. One of the primary sources of pathogen transmission is the improper management of bedding in these confined spaces. Once birds are introduced, bedding accumulates excreta, feed waste, feathers, broken eggs, and residual bedding as it transitions to litter. Potential pathogens include *Clostridium,* pathogenic *Escherichia coli, Staphylococcus, Campylobacter, Enterococcus,* and *Salmonella*. Pathogens in poultry litter can pass between generations and within flocks, as recontamination is a concurrent issue in these environments. The ability of bacterial pathogens to persist in litter, survive between production cycles, and serve as a continued source of flock reinfection will be described in this review. Poultry house and flock management strategies that may have an impact on pathogen survival and dissemination will also be discussed in this review.

## Introduction

To meet increasing demand for poultry meat, commercial broiler production has adopted intensive production systems characterized by large flock sizes, high stocking densities, and rapid turnover rates (Dittoe et al., 2020; Wythe et al., 2023). This intensification of live bird production, in turn, leads to the accumulation of waste materials, including litter bedding, manure, and other byproducts (Edwards and Daniel, 1992; Wang et al., 2025; Ricke et al., 2026b). Chickens continuously deposit intestinal microorganisms into litter via fecal shedding while also being exposed to these microorganisms through pecking behavior, dust inhalation, and direct contact with contaminated surfaces (Nodar et al., 1990; Cressman et al., 2010; Pal et al., 2021). The used litter, once removed from the poultry house, can be applied as fertilizer or converted into biogas (Nahm, 2007; Bolan et al., 2010; Wang et al., 2025). However, because this practice is cumulative, poultry litter can become a microbial reservoir that continually exposes birds to environmental emissions and microorganisms, including various pathogenic species (Cressman et al., 2010; Wang et al., 2025). Consequently, while these practices improve production efficiency, they also create environmental conditions that favor the accumulation and transmission of microorganisms. This occurs within poultry houses as well as beyond the poultry house, depending upon the endpoint for litter disposal and potentially increase the risk for dissemination of antibiotic resistance (Wadud et al., 2012; Chen and Jiang, 2014; Yang et al., 2021; Efriem et al., 2023; Wang et al., 2025).

Although poultry litter plays an important role in poultry house management, it also serves as a variable source of pathogens that can potentially persist in the poultry house environment (Terzich et al., 2000; Gomes et al., 2022; Plumblee Lawrence et al., 2022; Wang et al., 2025). Improper litter management can promote the establishment of pathogens, reinfect the flock, and pose risks to animal health, production efficiency, food safety, and public health (Wang et al., 2025). The objective of this review is to discuss the formation and bacterial pathogen ecology of poultry litter and the persistence, transmission, and control of major litter-associated pathogens (see Fig. 1 for overview). Emphasis is also placed on poultry house and flock management strategies that may impact microbial pathogen persistence.

**Fig. 1:**
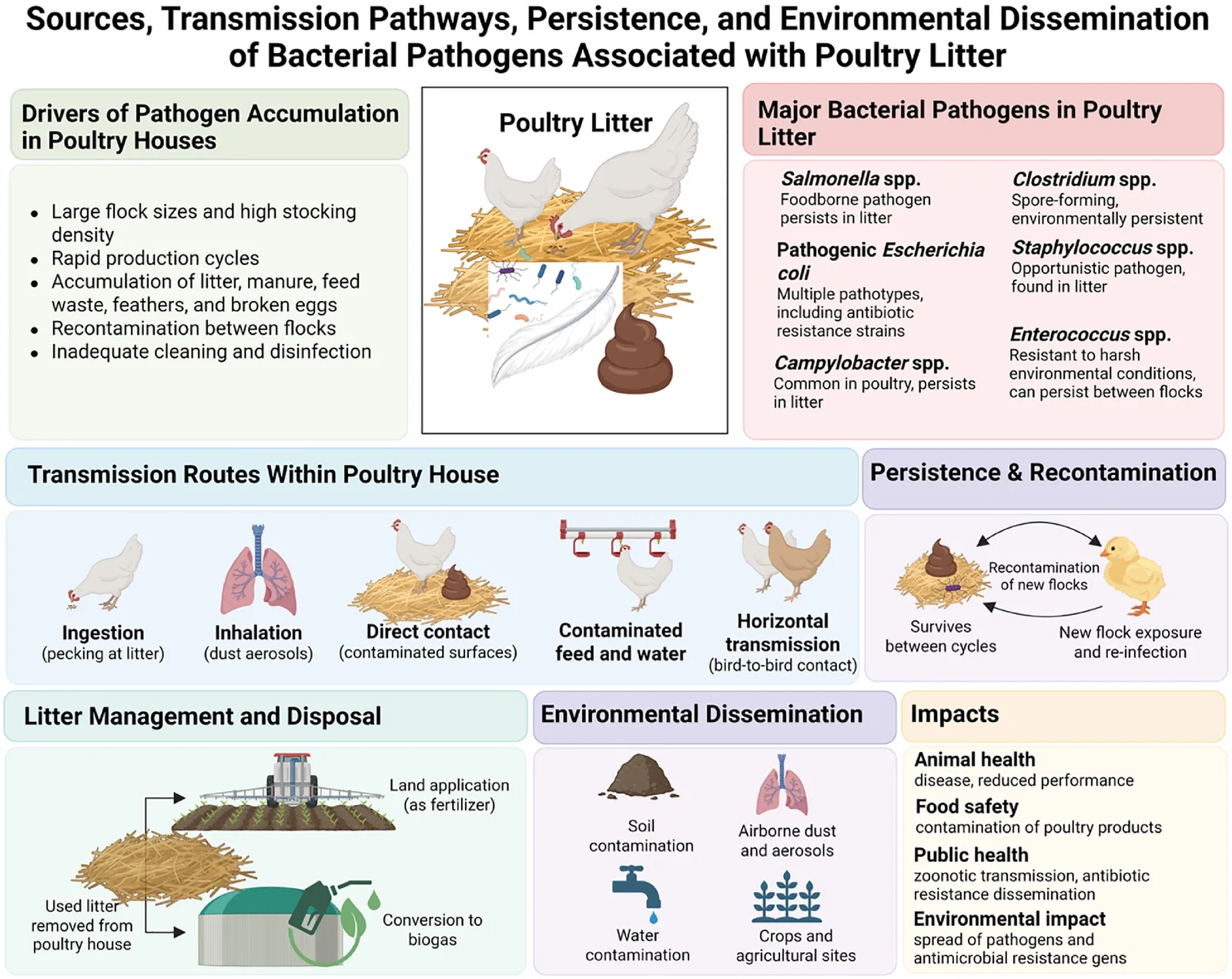
Schematic overview of the factors contributing to bacterial pathogen accumulation, transmission, persistence, and dissemination within and beyond poultry production environments. High stocking densities, rapid production cycles, organic matter accumulation, and incomplete cleaning and disinfection can promote the buildup of microorganisms in poultry litter. Major bacterial pathogens associated with poultry litter include *Salmonella* spp., pathogenic *Escherichia coli, Campylobacter* spp., *Clostridium* spp., *Staphylococcus* spp., and *Enterococcus* spp. Potential transmission routes include ingestion of contaminated litter, inhalation of dust and aerosols, direct contact with contaminated surfaces, exposure to contaminated feed and water, and horizontal transmission between birds. Pathogen survival in residual litter and the poultry house environment may contribute to recontamination during subsequent production cycles. Following litter removal, handling, storage, land application, and processing may facilitate pathogen dissemination into surrounding environments through contaminated soil, water, airborne dust, and agricultural systems, depending on management practices and treatment effectiveness. These processes have implications for poultry health and productivity, food safety, public health, and environmental contamination, including the potential dissemination of antimicrobial-resistant bacteria and resistance genes. Created in BioRender. Olson, E. https://BioRender.com/loymny4.

## The major bacterial players in poultry litter

Bedding is the unused poultry house floor substrate applied before bird placement, while litter is the resulting mixture of bedding, excreta, organic debris, moisture, and microorganisms that accumulate during poultry production (Diarra et al., 2021; Gomes et al., 2022; Ricke et al., 2026b). Poultry litter represents a microbial ecosystem that develops following bird placement and evolves throughout the production cycle (Bolan et al., 2010; Wang et al., 2025; Ricke et al., 2026b). Clean bedding materials are most often composed of wood shavings, rice hulls, cereal straw, husks, and paper clippings, among others (Swain and Sundaram, 2000; Bodie et al., 2019; Liegalz et al., 2025). As birds deposit feces, feathers, broken eggs, feed residues, and moisture, bedding undergoes substantial physical, chemical, and microbiological changes, ultimately becoming litter (Edwards and Daniel, 1992; Kelley et al., 1996; Tasistro et al., 2004; Wang et al., 2025). The microbial composition and microbial activities occurring in poultry litter are influenced by numerous factors, including but not limited to number of flocks raised on litter, litter moisture, ventilation, temperature, and litter re-use (Chinivasagam et al., 2010a; Wilkinson et al., 2011; Brink et al., 2022; Ivulic et al., 2022; Gomes et al., 2022; Oladeinde et al., 2023). Litter re-use versus replacement in commercial broiler production is highly variable. In general, litter is commonly recycled through several broiler production cycles before it is completely removed (Coufal et al., 2006). This can range from as few as two flock production cycles to several years (Coufal et al., 2006). As birds continuously shed microorganisms through fecal excretion, the litter becomes populated by a wide variety of microorganisms, including several pathogenic antibiotic-resistant species (Nodar et al., 1990; Locatelli et al., 2017; Ngogang et al., 2021; Gomes et al., 2022; Wang et al., 2025). Moreover, this biological activity occurring within litter has important implications for pathogen persistence and establishment, potentially leading to public health concerns (Gomes et al., 2022). Consequently, poultry litter serves as a significant reservoir for microorganisms that influence bird performance, disease susceptibility, and the overall microbiological status of poultry production systems.

Initially, the microbial composition of fresh litter comprises microorganisms already present in the environment and in the bedding material itself. After 7-day exposure to birds, fresh bedding originating from raw materials such as straw, wood products, and plant-derived substrates commonly harbored aerobic mesophilic bacteria, environmental fungi and yeasts, as well as important genera such as *Staphylococcus, Escherichia, Listeria, Salmonella*, and *Candida* (Gontar et al., 2022). However, their abundance likely varies with source material, storage conditions, and processing methods. The primary contributors to the shift from bedding to litter are the poultry gastrointestinal tract (GIT) microbiota and the poultry housing environment (Lu et al., 2003; Lovanh et al., 2007; Wang et al., 2016). The chicken is considered the most important contributor to the poultry litter microbial community with chicken fecal material being a primary source along with the microorganisms derived from feathers or exfoliated skin epithelial cells also becoming part of the litter ecosystem (Kubasova et al., 2022). As microorganisms become attached to the bird's intestinal lining and are shed, litter communities are dominated by microorganisms originating from the GIT (Cressman et al., 2010). As the resulting fecal buildup occurs, the dissemination of intestinal pathogenic bacteria such as *Salmonella, Campylobacter,* APEC (avian pathogenic *Escherichia coli*)*, Staphylococcus* spp., *Enterococcus* spp., and *Clostridium perfringens* among others can occur through the fecal-oral route followed by potential proliferation leading to detectable contaminants emerging in the poultry litter (Cressman et al., 2010; Broom, 2017; Locatelli et al., 2017; Fancher et al., 2020; Plumblee Lawrence et al., 2022; Wang et al., 2025). Similar observations indicate that the original bedding microbiota declines following bird introduction and is replaced by microorganisms associated with a newly developing litter environment, largely due to continuous deposition of chicken feces (Kubasova et al., 2022; Gontar et al., 2022).

Poultry litter thus functions as both a recipient and source of microorganisms (Fig. 1). Birds continuously deposit microorganisms into litter while being exposed to microorganisms through several sources including dust exposure and direct contact with fecal material (Chinivasagam et al., 2009; Cressman et al., 2010; Pal et al., 2021). This relationship creates continuous microbial exchange between the bird and its surroundings, contributing to the establishment and maintenance of microbial populations during live bird production. This can even have longer-term impacts, depending on the contribution of litter microorganisms to the underlying soil microbial consortia of the poultry house floor (Crippen et al., 2019). Studies on poultry litter from multiple geographical regions and other sources have identified several pathogenic and/or potentially pathogenic bacteria, including *Salmonella, E. coli, Campylobacter, Staphylococcus, Clostridium*, among others (Terzich et al., 2000; Plumblee Lawrence et al., 2022; Wang et al., 2025). However, more comprehensive assessments are difficult to summarize, as several factors, including detection methodology, sampling location, and geography, can contribute to observed variations (Locatelli et al., 2017; Winkler et al., 2017; Plumblee Lawrence et al., 2022). The following sections discuss some of the more extensively documented bacterial pathogens associated with poultry litter that are listed in Table 1.

**Table 1:** Major bacterial pathogens associated with poultry litter and their ecological interpretation.

**Pathogen/group**	**Main poultry/litter relevance**	**Litter conditions that favor concern**	**Ecological interpretation**	**Practical implication**
*Salmonella*	Major foodborne pathogen; can persist in litter, feed, water lines, and poultry house environments	Wet/caked litter, manure-rich zones, biofilms, reused litter, late-flock buildup	Persistence-associated pathogen; reflects long-term survival, recontamination potential, and moisture-protected niches	Target water-line sanitation, litter drying, caked litter removal, feed/water biosecurity, and source tracking
*Campylobacter jejuni/C. coli*	Major foodborne pathogen; colonizes birds asymptomatically and spreads rapidly after flock introduction	Moist fecal-rich litter, contaminated litter, high humidity, reused litter, bird pecking/contact exposure	Rapid flock-spread organism; litter may act more as a short-term source/sink after colonization than a long-term reservoir	Monitor late grow-out, reduce moist refuges, control pests/water sources, and pair litter testing with bird/carcass testing
*Clostridium perfringens*	Associated with necrotic enteritis, wet litter, diarrhea, and disease-related shedding	Nutrient-rich, wet, compacted, low-oxygen/caked zones; disease or enteric disturbance events	Gut–litter anaerobe/opportunist; indicates organic matter turnover, oxygen limitation, and disease-linked microbial amplification	Focus on enteric health, moisture prevention, caked litter management, and monitoring during necrotic enteritis/coccidiosis events
APEC / *E. coli*	Opportunistic pathogen linked with colibacillosis, respiratory/systemic infection, and antimicrobial resistance	Wet litter, fecal buildup, dust, poor water/feed hygiene, reused litter	Fecal indicator and opportunistic pathogen; maps manure deposition, moisture hotspots, and hygiene breakdown	Use as an indicator organism with moisture, dust, and ammonia measurements; improve litter dryness and biosecurity
*Enterococcus*	Common gut/litter organisms; some species linked with spondylitis, septicemia, lameness, and antimicrobial resistance	Fecal-rich litter, reused/stored litter, wet zones, disturbed microbial communities	Fecal/opportunistic indicator; useful for tracking bird deposition, persistence, and AMR reservoirs	Combine culture/qPCR with resistance screening; consider how broad amendments affect beneficial vs. opportunistic taxa
*Staphylococcus*	Common in litter, dust, skin, feathers, water, and equipment; some species cause bumblefoot, arthritis, osteomyelitis	Dusty litter, skin injury, high contact areas, stored litter, poor sanitation	Ubiquitous environmental/skin-associated organism; reflects bird-contact surfaces and injury-related exposure risk	Reduce skin/footpad damage, manage litter moisture and ammonia, remove caked litter, and disinfect equipment

### Clostridium perfringens: the anaerobic opportunist of wet litter

*Clostridium perfringens* is a spore-forming anaerobic bacterium that is distributed throughout poultry production environments (Fathima et al., 2022). Several factors and predisposing conditions can lead to *C. perfringens* infection in birds including wet litter and high protein diets along with increased bird flock density, immunological status of the bird and environmental conditions such as heat stress (Broom, 2017; Fathima et al., 2022). The organism is ubiquitous within these ecosystems in part due to its ability to form environmentally resistant spores, mostly associated with litter, that enable survival under harsh conditions and promote persistence in environments such as poultry houses for extended periods (Van Immerseel et al., 2004, 2009; Liggins et al., 2023). Although present as a species of normal intestinal microbiota, pathogenic strains of *C. perfringens* can produce a range of toxins, enzymes, and virulence factors that support disease development (Cooper et al., 2010; Li et al., 2016, 2017; Broom, 2017). More than 20 toxins have been identified, with alpha toxin (CPA) and necrotic enteritis B-like (NetB) toxin among the more extensively studied toxins (Keyburn et al., 2008; 2010; Cooper et al., 2010; Coursodon et al., 2010; Fathima et al., 2022). Beyond its significance as a poultry pathogen, *C. perfringens* is also a relevant foodborne bacterial microorganism found in humans, with poultry products representing a common source of transmission (Grenda et al., 2023). Due to its environmental persistence, widespread distribution, and pathogenic potential, *C. perfringens* remains an important bacterial species influencing poultry health and litter microbial ecology.

*Clostridium perfringens* promotes enteric disease by causing necrotic enteritis, a debilitating disorder common in broiler production worldwide, characterized by intestinal necrosis, impaired nutrient absorption, reduced feed intake, poor feed conversion, decreased growth performance, and increased mortality (Lee and Lillehoj, 2022). It is estimated that necrotic enteritis, caused primarily by type A *C. perfringens,* results in a loss of billions of dollars per year in the international poultry industry due to production losses and control measures (Moore, 2016; Broom, 2017; Fathima et al., 2022). As stated earlier, NetB toxin is a primary contributor to the formation of necrotic enteritis pathogenesis within broilers, as NetB is a toxin that damages intestinal epithelial cells by creating transmembrane pores within cell membranes, disrupting ion homeostasis, and consequently causing cell swelling, lysis, and tissue necrosis (Keyburn et al., 2010; Popoff, 2014; Fathima et al., 2022). Furthermore, as the disease progresses, extensive epithelial damage and lesion formation in the small intestine result in tissue necrosis, exposing vulnerable tissues to bacterial colonization and causing the intestinal mucosa to become inflamed and covered with characteristic necrotic plaques, reducing digestive efficiency and overall bird performance (Fathima et al., 2022). As the disease progresses, damaged intestinal tissues, increased bacterial populations, and shed epithelial cells are excreted in chicken feces, introducing substantial levels of *C. perfringens* into the litter environment.

Once ingestion and exposure to *Clostridium* species spores, particularly the most common type A, *C. perfringens,* occur*,* necrotic enteritis begins to impact birds. An additional consequence of this disease is the development of watery stools and diarrhea in birds, promoting the formation of wet litter and the deposition of more *C. perfringens* spores and particles (Fancher et al., 2020). Inappropriate nutrition and other causes of wet litter can, in turn, promote the growth, survival, and transmission of microorganisms, including *C. perfringens* and *E. coli* (Eriksson de Rezende et al., 2001; Collett, 2012; Fancher et al., 2020). Beyond pathogen persistence, excessive litter moisture is associated with the development of footpad dermatitis (FPD), a condition characterized by lesions and ulceration of the footpad resulting from direct contact with wet litter and high ammonia levels (Dunlop et al., 2016). This litter moisture promotes FPD and other forms of skin damage such as hyperkeratosis and necrosis of the epidermis and potentially eventual ulceritis and inflammatory responses (Kjaer et al., 2006). Poor litter conditions and skin lesions are recognized risk factors for gangrenous dermatitis (GD), a severe clostridial disease most seen with *Clostridium perfringens* type A and *Clostridium septicum* (Gornatti-Churria et al., 2018). Damaged skin provides a route of entry for infection, while wet litter and elevated ammonia concentrations further support disease development, stress, immune suppression, and chemical burns, creating conditions that favor bacterial invasion and rapid tissue necrosis (Kjaer et al., 2006; Gornatti-Churria et al., 2018). Dunlop et al. (2016) recommended practices that suppress litter moisture to prevent the development of litter-associated diseases, including footpad dermatitis and gangrenous dermatitis.

## Avian pathogenic *E. coli: persistence and antibiotic resistance*

Avian pathogenic *E. coli* (APEC) is an important pathogen responsible for the adoption of colibacillosis in broilers, layers, and breeders (Watts and Wigley, 2024). Avian pathogenic *E. coli* is unique in that it can act as both a primary and opportunistic pathogen, causing respiratory, systemic, and even reproductive infections, including airsacculitis, cellulitis, pericarditis, peritonitis, and more (Watts and Wigley, 2024; Perez et al., 2025). As a result, higher mortality rates, reduced productivity, and increased carcass disposal are observed, leading to increased economic losses (Watts and Wigley, 2024). In addition, the ability of certain APEC strains to become extraintestinal, develop antimicrobial resistance, and survive in litter environments makes them especially threatening to poultry production settings (Khong et al., 2023; Watts and Wigley, 2024).

When Khong et al. (2023) isolated *E. coli* from litter collected at day 53 of a broiler trial they detected resistance to 10 antimicrobials along with 23 antimicrobial and heavy metal resistance genes, O serogroups, and avian pathogenic *E. coli* minimal predictor genes. Over 50% of the *E. coli* isolates were resistant to cephalothin followed by lower percentages to tetracycline, streptomycin, ampicillin, colistin, sulphonamides, and imipenem (Khong et al., 2023). In addition, they noted multidrug resistance to at least 3 antimicrobials in over 20% of isolates. Velhner et al. (2020) also observed multidrug resistance in *E. coli* isolates from a farm experiencing an avian pathogenic *E. coli* colisepsis incident*.* They detected multidrug resistant *E. coli* in over 70% of the cloacal isolates and 100% of the litter isolates from the 5 poultry houses that they sampled. They noted that the highest resistance of the *E. coli* isolates was to ampicillin and tetracycline. However, when the poultry isolates and litter isolates resistant to 7 antibiotics of different classes were identified using pulsed field gel electrophoresis genotyping they were not clonal but were genetically diverse. Based on the presence of conjugative plasmids harboring virulence genes, multiple drug resistance genes and class 1 integrons, Velhner et al. (2020) proposed that horizontal gene transfer may be occurring leading to the dissemination of multidrug resistance among different isolates. They suggested that litter could serve as a source of multidrug-resistant genes that persist in the farm environment and be transmitted to other *E. coli*. The horizontal transfer of resistance genes indicates that poultry litter not only harbors pathogenic strains but would potentially support the emergence and spread of antimicrobial-resistant genotypes. Poultry house flooring design is also a likely factor. When Chuppava et al. (2019) compared litter covered flooring versus housing with elevated slat platform on multi-drug-resistant commensal *E. coli* they concluded that bird to bird contact could be more of a reservoir than the litter-excreta matrix.

The ability of *E. coli* to persist over extended periods of time in poultry house environments remains a factor that should be further considered. Chinivasagam et al. (2010b) detected *E. coli* in both single use and re-used poultry litter samples from Australian poultry farms leading them to suggest that *E. coli* may possess the potential to survive in poultry litter for extended periods of time. Mo et al. (2021) demonstrated that extended-spectrum cephalosporin resistant *E. coli* and their plasmids could persist over 6-months in Norwegian broiler houses and that some genetic variants could persist between production cycles. This is supported by Daehre et al. (2018) who concluded that extended-spectrum beta-lactamase and AmpC beta-lactamase Enterobacteriaceae could potentially be transferred from one flock to the next via contaminated poultry houses. The impact of bird placement is a likely factor. When Chinivasagam et al. (2009) followed *E. coli* levels over time in poultry farms they reported that *E. coli* increased rapidly after chick placement but, after a slight decrease, stabilized over 50 days. Furthermore, aerosol *E. coli* populations were not detectable until chick placement. Survival of *E. coli* in poultry house environments may be strain dependent. For example, Nguyen et al. (2022) compared the survivability of airborne dust and settled dust *E. coli*, with *E. coli* in poultry litter using laboratory scale test chambers. The resulting values for each sample type were half-life times for airborne *E. coli* of 6 minutes versus hours for settled *E. coli* (nearly 10 hours) and poultry litter (nearly 16 hours). However, earlier work by Nicholson et al. (2005) demonstrated that *E. coli* O157 could survive in stored livestock manure slurries and dirty water for up 6 months. More research needs to be done to compare different *E. coli* seorvars/strains and survivability in litter to determine if differences exist and whether external factors such as litter amendments influence survival kinetics. In addition, based on the evidence of potential transmission of antibiotic-resistant populations from one flock to the next, efforts to introduce hygienic measures at the farm level appear to be important as suggested by Daehre et al. (2018).

### Campylobacter: rapid horizontal transmission and the role of litter

Campylobacteriosis is a major bacterial foodborne disease worldwide, primarily caused by *Campylobacter jejuni* and *Campylobacter coli* (Khairullah et al., 2024). Poultry serve as a major reservoir of these pathogens, and unlike some enteric pathogens, *Campylobacter* can colonize the GIT of broiler chickens without causing clinical disease or noticeable symptoms, allowing infected flocks to act as asymptomatic carriers throughout production (Horrocks et al., 2009; Awad et al., 2015; Indikova et al., 2015; Zainol et al., 2024). Horizontal transmission of *Campylobacter* occurs from already colonized birds and environmental sources (Bundurus et al., 2024). Once susceptible birds are exposed colonization in a flock can be fairly rapid. In an Australian study, van Gerwe et al. (2009) quantified *C. jejuni* field data for over 5 years to estimate a transmission rate of over infection per infectious per day ultimately resulting in a prevalence of 95% within a flock within 4 to 7 days after appearing in the first bird starting at age 21 days. Age of birds appears to be important as Connerton et al. (2018) reported that 20- day old birds exhibited detectable *Campylobacter* cecal colonization within 2 days of exposure, versus 6-day old birds which required 9 days after initial infection. Increased *Campylobacter* colonization has been associated with insufficient farm hygiene, reduced flock replacement period, proximity to other farm animals, rodents and insects, seasonal changes, and partial flock depopulation (Sibanda et al., 2018).

Litter has been identified as a potential *Campylobacter* reservoir as well (Sibanda et al., 2018). When Chinivasagam et al. (2009) followed *Campylobacter* levels over a commercial production cycle they detected *Campylobacter* late in the production cycle from litter samples but not in aerosols. Follow up studies by Chinivasagam et al. (2010b) using most probable number (MPN) quantitation detected low levels of less than 100 MPN for most of the positive litter samples collected from Australian farms at the end of a production cycle. However, they concluded that this may have been because of the fragility and subsequent loss of viable cells. In a later study, Chinivasagam et al. (2016) compared 3 different litter re-use management approaches (new litter for each production cycle; partial litter re-use; and complete litter re-use) on *Campylobacter* occurrence in broiler ceca and litter from Australian poultry farms for 6 production cycles over a 2-year period. Using a combination of MPN for quantitation and real-time polymerase chain reaction (PCR) for confirmation and species identification, they concluded that *Campylobacter* appearance and levels did vary by farms, production cycles, and final bird pick-ups. However, regardless of whether *C. jejuni* or *C. coli* were prevalent in ceca or litter, the management of litter did not directly impact this outcome (Chinivasagam et al. (2016). Bailey et al. (2022) observed rapid dissemination of *C. jejuni* among birds and the litter after inoculating a subset of seeder birds with marker strains of *C. jejuni*, but after a 19-day downtime no *C. jejuni* were detected in the 2nd flock when placed on this previously contaminated litter. In a longitudinal study, Chinivasagam et al. (2024) followed *Campylobacter* spp. from the farm to the processing plant from commercial poultry operations involving either conventional full-clean-out, conventional litter re-use, free-range litter full-cleanout, or free-range litter re-use. They concluded that despite these differences in farm management neither cecal levels nor carcass levels of *Campylobacter* were impacted by the respective litter production practice.

The interaction between litter and the physiological status of *Campylobacter* appears to be relatively complex and in turn may impact recovery of culturable *Campylobacter*. For example, *in vitro* studies conducted by Smith et al. (2016) reported that *C. jejuni* survived longer in fresh feces than used broiler litter and was enhanced under microaerophilic versus aerobic incubation conditions. When Oladeinde et al. (2023) collected samples for farms with peanut hull-based litter they reported that recovery of *Campylobacter* increased with litter pH levels above 7 and other environmental factors likely played a role as well. However, they concluded that that the environmental interactions remained complex leading to difficulties for interpretation and comparisons across studies in part due to the viable nonculturable nature of *Campylobacter*. This is supported by the intricate growth requirements of *Campylobacter* that continues to challenge developing culture media that will support optimal growth (Olson et al., 2024a; Ricke et al., 2026a). Despite culture difficulties, the resulting *Campylobacter* isolates can exhibit characteristics associated with litter management. Woyda et al. (2023) further characterized *Campylobacter* isolates from their previous study (Oladeinde et al., 2023) employing antimicrobial testing and whole genome sequencing leading to the conclusion that house environment and management practices were potentially selective on the presence of antimicrobial resistance and virulence properties. Gharbi et al. (2023) identified high levels of antimicrobial and virulence genes from *Campylobacter* isolates after farm disinfection and suggested that this practice could lead to persistence and cross contamination of incoming flocks.

In addition to environment and house management differences, the presence of other organisms in the litter ecosystem may influence *Campylobacter* levels. Valeris-Chacin et al. (2021) collected boot swab samples once from poultry barns that housed birds between 3 weeks of age to the end of the grow-out period and conducted 16S rDNA microbiome sequencing in parallel with *C. jejuni* and *C. coli* isolation. They observed positive associations between *Campylobacter, Clostridium* and *Anaerostipes* versus negative associations with *Bifidobacterium, Anaerosporobacter*, and *Stenotrophomonas*. In a follow up study Valeris-Chacin et al. (2022) tracked longitudinal patterns of *Campylobacter* and the litter microbiome from 1 to 2 weeks of placement to the end of the production cycle. At 6 weeks of age a negative correlation was observed between *Campylobacter* and genera *Bogoriella* and *Pseudogracilibacillus*. Relationships were also noted at earlier bird ages. At 2 and 4 weeks of age, Bayesian networks analyses revealed negative associations between *Campylobacter* and two litter microbiome bacterial genera, *Ornithinibacillus* and *Oceanobacillus*. Collectively these results suggest potential interactions between the litter microbial community and *Campylobacter* occurrence. This is consistent with the *in vitro* cecal incubation studies by Olson et al. (2024b; 2025) indicating that complex metabolic interactions and nutrient cross-feeding may be occurring between *C. jejuni* and the cecal microbiota. Similar scenarios could occur in litter microbial ecosystems and may help explain the variable recoveries of *Campylobacter* spp. from litter. The presence of antagonistic bacterial members of the litter microbiome populations has been suggested as potential probiotic candidates and there has been general interest in developing probiotics specific for *Campylobacter* (Deng et al., 2020; Valeris-Chacin et al., 2021; 2022). Other biological factors such as *Campylobacter* bacteriophage have also been identified in poultry litter as a potential control measure and bacteriophage have been historically explored as potential interventions for *Campylobacter* in poultry (Chinivasagam et al., 2020; Olson et al., 2022b). Litter management, effective sanitation practices, and the implementation of potential control measures such as bacteriophage and probiotics offer the potential to reduce the risk of *Campylobacter* transmission within poultry houses. However, optimizing *Campylobacter* interventions requires an understanding of the complex metabolic interrelationships between *Campylobacter* and the litter microbial consortia.

### Staphylococcus: a contact-associated resident of litter, dust, skin, and feathers

*Staphylococcus* spp. are among the most common microbial residents of production environments and are perpetually found within litter, dust, water, and farm equipment (Szafraniec et al., 2022). Staphylococci are considered ubiquitous members of poultry microbiota and persist throughout consistent production cycles (Szafraniec et al., 2022; Combar et al., 2026). A wide range of *Staphylococcus* species have been isolated from poultry, including *S. epidermidis, S. xylosus, S. cohnii, S. lentus, S. saprophyticus, S. sciuri, S. gallinarum*, and *S. aureus* (Szafraniec et al., 2022; Combar et al., 2026). While many staphylococcal species maintain commensal relationships with their hosts, opportunistic species, such as *S. aureus and S. lentus* have been associated in case studies with outbreaks of arthritis, synovitis, and osteomyelitis (Matos et al., 2024). Staphylococcal infections most often occur after damage to skin or mucosal barriers, providing a route for bacteria from the surrounding environment to invade tissues (Combar et al., 2026). Infections are commonly facilitated by skin injuries, inflammation of mucosa, immunosuppressive conditions, or even unhealed navels in newly hatched chicks, all of which compromise the host’s defense and increase susceptibility to disease (Szafraniec et al., 2022; Combar et al., 2026). Conditions associated with *Staphylococcus* include arthritis, osteomyelitis, bumblefoot, chondronecrosis with osteomyelitis, and skeletal disorders that ultimately produce reduced productivity and lameness (Szafraniec et al., 2022).

*Staphylococcus* spp. have been associated with poultry house environments with the genus consistently observed throughout grow-out and effectively one of the more commonly isolated groups of organisms from poultry house air samples (Chinivasagam et al., 2010a; Szafraniec et al., 2022). These observations are not surprising given that the *Staphylococcus* spp. originated from the chickens (Chinivasagam et al., 2010a). Given the close association with birds, poultry litter is also a likely reservoir for staphylococci. When Terzich et al. (2000) screened litter samples from commercial broiler houses from 12 different geographical regions in the U.S. they identified staphylococci as the most frequent inhabitant of fresh litter for all regions but with statistical differences in total numbers by region. In a later study, Adhikari et al. (2020) conducted a survey of litter boot swab samples from commercial turkey farms using 16S rRNA gene sequencing and identified *Staphylococcus* as one of only 4 core genera present in all litter samples. Despite the fact that staphylococci proliferation and exposure can be traced to litter, its presence originating from the poultry’s natural microbiome presents an issue when formulating biosecurity practices aimed at minimizing *Staphylococcus*-induced diseases.

The presence of staphylococci in litter is further complicated by the fact that not only can several species potentially cause disease in poultry but they may carry multiple antibiotic resistance genes (Szafraniec et al., 2022). For example, Blżażejewska et al. (2022) screened poultry litter from poultry farms in Poland and isolated pathogenic *Staphylococcus lentus* that possessed resistance to multiple antibiotics. Extended storage and treatment of poultry litter do not appear to offer effective mitigation of litter staphylococci. Anaerobic digestion of tetracycline spiked poultry litter succeeded in decreasing tetracycline concentration but increased the level of total bacteria, *E. coli*, enterococci, and *Staphylococcus aureus* as well as *tet* genes and methicillin resistant genes (Agga et al., 2020). Furthermore, antimicrobial-resistant staphylococci have been shown to persist in stored poultry litter despite prolonged storage over 120 days (Graham et al., 2009). More recently, Gupta et al. (2021) reported persistence of mostly nonpathogenic *Staphylococcus* spp. in litter stockpiles stored over several weeks but low levels of *S. aureus* was also detected at later timepoints. Eradication of staphylococci may not be either practical nor feasible, as birds continually generate these organisms through feces, skin, and feathers as a part of their constant relationship with staphylococci (Combar et al., 2026). Therefore, devising intervention strategies for litter staphylococci remains a management challenge and likely require more studies focused on litter conditions that support sustained versus diminished levels of staphylococci. In addition, determining if differences occur among disease causing species versus non-disease staphylococci and factors impacting antibiotic resistance would potentially contribute to devising approaches for minimizing the detrimental properties associated with poultry litter staphylococci.

### Enterococcus: commensal, opportunist pathogen, and antimicrobial resistance reservoir

The genus *Enterococcus* consists of Gram-positive, facultatively anaerobic, non-spore-forming lactic acid bacteria within the phylum Firmicutes (García-Solache and Rice, 2019). Numerous *Enterococcus* spp. have been described from diverse natural niches, including the GIT of animals and humans, soil, water, plant material, insects, fermented foods, and food animal production environments (García-Solache and Rice, 2019; Kalode and Patil, 2023). Their ability to produce bacteriocins has made some *Enterococcus* spp. attractive as a source of antimicrobial compounds and as potential probiotics (Hanchi et al., 2018). For example, some *E. faecalis* and *E. faecium* strains have been employed as probiotics in broilers and laying hens to improve performance and increase egg production (Park et al., 2016; Neveling et al., 2020). However, these applications remain controversial due to the ability of enterococci to acquire virulence factors and antibiotic resistance (Krawczyk et al., 2021). Consequently, pathogenic enterococci are a public health concern with the two most prominent species, *E. faecalis* and *E. faecium* being responsible for urinary tract infections, bloodstream infections, and endocarditis in humans (Lu et al., 2025). An additional challenge is that these enterococci can, not only easily acquire antibiotic resistance via chromosomal mutations and horizontal gene transfer, but are now exhibiting resistance to antibiotics that would be considered “last-line” antibiotics such has vancomycin among others (Lu et al., 2025).

*Enterococcus faecalis* and *E. faecium* are the most frequently recovered and best-characterized species in broiler operations (Souilard et al., 2022; Reynolds et al., 2024). These two species typically dominate the GIT in 1-day old chicks (Devriese et al., 1991). These two species have been isolated from various skin lesions in poultry as well as endocarditis and amyloidosis (Souilard et al., 2022). To a lesser extent, *E. hirae, E. durans, E. avium*, and *E. cecorum* have also been isolated from poultry systems, but their habitat function and clinical significance differ substantially (Devriese et al., 1991). However, since initial outbreaks were documented in 2002, *Enterococcus cecorum* has received increased attention as a concern in poultry production (Jung et al., 2018). Once considered commensal members of the avian GIT, pathogenic lineages of *E. cecorum* have been recognized as causative agents of enterococcal spondylitis, vertebral osteomyelitis, septicemia, and lameness in commercial broilers (Borst et al., 2017; Jung et al., 2018). Initially *E. cecorum* occur as commensal strains at minimal levels in the young chick GIT up to 3 to 4 weeks before becoming the dominant member of enterococcal and streptococcal populations in 12 week old birds whereas pathogenic strains colonize the GIT of chicks as young as 7 days but are replaced by commensal *E. cecorum* as the bird ages (Jung et al., 2018).

Antimicrobial-resistant enterococci and resistance determinants have been shown to persist in stored poultry litter, and in turn, temporarily enrich total and antimicrobial resistant enterococci in poultry litter amended soils (Graham et al., 2009; Agga et al., 2025). However, reports on occurrence of *Enterococcus* spp. in poultry litter have been somewhat inconsistent. Early studies by Kuntz et al. (2004) using enterococci selective media detected *E. faecalis* in nearly half of the fresh broiler feces but rarely in the poultry litter from University of Georgia (Athens, GA) poultry farms. This led them to suggest that the litter matrix environment is likely to select against *E. faecalis.* However, this may be somewhat dependent on the methodology employed for identification as well as the geographical origin of the samples. For example, Graham et al. (2009) monitored microbial populations in poultry litter stored over 120 days from U.S. commercial farms and using multiplex PCR identified several antibiotic resistant *Enterococcus* spp. of which *E. faecalis* (70%), *E. faecium* (14%), *E. casseliflavus* (4%) were identified along with 12% unclassified *Enterococcus* spp. Efriem et al. (2023) using MALDI-TOF Mass Spectrometry (MS) for identification of *Enterococcus* spp. concluded that *E. faecium* was more dominant than *E. faecalis* in litter collected from Israeli broiler farms. Vellner et al. (2024) conducted a survey of 40 Serbian poultry farms and using PCR or MALDI-TOF MS identified *E. faecium* from 42.5% of the farms followed by *E. faecalis* (37.5%), *E. hirae* (15%), *E. durans* and *E. thialandicus* (2.5%). In addition, resistance to erythromycin, streptomycin, fluoroquinolones, and tetracyclines among the frequently used antibiotics was detected with multidrug resistance also occurring. Other environmental factors may play a role as well. For example, based on microbiome analyses, Gupta et al. (2021) reported a positive correlation in occurrence of *E. faecium* and *E. faecalis* in litter stockpiles with oxygen levels. Environmental reservoirs for *E. cecorum* have not been well established. Jung et al. (2018) suggested that a farm-associated reservoir is possible but had yet to be identified. More recently, Tessin et al. (2024) conducted a comprehensive environmental survey of poultry barns housing clinically healthy broilers and *E. cecorum* in fecal samples were significantly greater and generally occurred towards the end of the production cycle.

Interpretation of *Enterococcus* spp. occurring in poultry litter is somewhat complicated since some strains can also be used as probiotics (Hanchi et al., 2018). This suggests that altering the poultry GIT with biotic compounds, such as *E. faecium*, could alter the dynamics of litter composition. It would be interesting to determine if *Enterococcus*-based probiotics would behave as competitive exclusion cultures and limit *Enterococcus* pathogen prevalence and dissemination in litter. If that is the case, this also emphasizes that the application of broad-spectrum litter amendments must be carefully considered versus potential adverse impacts on beneficial litter microorganisms. In addition, given the pathogenic nature of some *E. faecium* strains it will be critical to develop identification methodology that clearly differentiates pathogenic versus probiotic *E. faecium*.

### *Salmonella*: the persistent reservoir pathogen

*Salmonella* is considered one of the more important foodborne pathogens associated with poultry production. The organism colonizes the GIT of poultry and can be transmitted both vertically and horizontally within hatcheries and poultry houses through litter, feed, water, pests, and the environment (Maciorowski et al., 2004; Park et al., 2008; Obe et al., 2023; Wang et al., 2025). Once established, *Salmonella* is difficult to eradicate and persists within birds and the production environment, resulting in continual recontamination and dissemination (Park et al., 2008). Overall, *Salmonella* infections in broilers and laying hens create economic and public health concerns for the industry by promoting production losses, requiring implementation of biosecurity practices, and being a threat to public health and food safety (Foley et al., 2011; O’Bryan et al., 2022; Obe et al., 2023; Gast et al., 2024). In live bird production poultry litter can serve as a potential reservoir for foodborne *Salmonella* serovars leading to persistence and potential infection of the flock. Furthermore, poultry litter can influence subsequent *Salmonella* persistence in soils, where it has been shown to survive for up to 4 months, although this may be strain-specific (Gu et al., 2019; Bardsley et al., 2021).

*Salmonella* has been a longstanding target of litter research due to its persistence and ability to survive in litter, feed, and water lines. Under favorable environmental conditions, *Salmonella* has been recovered from contaminated poultry litter more than 18 months after initial inoculation, demonstrating its ability to persist outside the host (Williams and Benson, 1978). This ability for long term survival is partly because *Salmonella* possesses numerous resistance and tolerance mechanisms for surviving environmental conditions associated with litter, such as exposure to organic fermentation acids, as well as thermal and cold stressors (Ricke, 2003; Dawoud et al., 2017; Dittoe et al., 2018; Ricke et al., 2018). Persistence is influenced by interactions between the organism and the litter environment, including moisture content, temperature, airflow, and physicochemical conditions that influence pathogen survival (Bodi et al., 2013; Biswas et al., 2019; Pal et al., 2021; Avidov et al., 2021). Persistence is also a function of different environmental interfaces interacting. For example, based on chicks inoculated with *S*. Typhimurium in a challenge study, Kwon et al. (2000) demonstrated that airborne *S*. Typhimurium could be detected within days after appearing in the litter. However, increasing litter moisture can decrease *Salmonella* prevalence in the dust originating from litter (Pal et al., 2021).

The ability of *Salmonella* to survive in dry re-used poultry litter is largely attributable to its physiological adaptations that enhance survival outside the host. Poultry litter conditions have been shown to influence stress responses in *Salmonella*. For example, Paudel et al. (2026) compared *S*. Infantis transcriptomic responses to the presence or absence of poultry litter and concluded that virulence genes were generally downregulated in litter while acid stress and anaerobic respiration genes were upregulated. Transcriptional profiles not only represent potential molecular mechanisms that may explain the ability of *Salmonella* to persist in poultry litter but could help to identify additional targets for litter amendments to more effectively eliminate *Salmonella*. Among these adaptations is resistance to osmotic and desiccation stresses, which enables the *Salmonella* to withstand the dry, hostile conditions outside the host increasing virulence responses and the ability to cause infection (Oliveira et al., 2026). *Salmonella* responds to desiccation conditions by reshaping cellular membranes and accumulating protective molecules ultimately leading to biofilm formation as a stress response (Oliveira et al., 2026). The overall desiccation resistance responses of *Salmonella* allow it to survive in environments such as dry poultry litter for extended periods and potentially resist other environmental stresses. When Chen et al. (2013) inoculated aged-litter with a 4 serovar *Salmonella* serovar combination that had been desiccation adapted for 24 hours they noted that desiccation adaptation increased thermal resistance when compared to non-desiccation adapted *Salmonella*. In a follow up study, Chen et al. (2015) demonstrated that storage time was a factor as desiccation-adapted *Salmonella* cells in fresh chicken litter were more susceptible to thermal inactivation than those in aged chicken litter. Finally, Chen and Jiang (2017) examined desiccation-adapted and rehydrated *S*. Typhimurium thermal resistance and gene expression in aged broiler litter. They detected rapid induction of desiccation adapted *S*. Typhimurium genes *rpoS, proV, dnaK*, and *grpE* and concluded that these genes may be involved with desiccation survival, with the *rpoS* gene potentially enabling cross protection against high temperatures.

Re-used litter may have indirect impacts as well on *Salmonella* persistence. When assessing risk factors associated with *Salmonella* and poultry litter, Volkova et al. (2011) concluded applying fresh wood shavings to top-dress or to completely replace the litter between flocks was more likely to result in greater detection of *Salmonella*. Chinivasagam et al. (2012) followed the progression of *Salmonella* serovars in commercial Australian poultry tunnel ventilated barns that practiced partial litter re-use. The litter re-use program described by Chinivasagam et al. (2012) involved windrowing litter at the end of the first broiler cycle, followed by barn cleaning, and dispersing the resulting windrowed litter in the grow-out section of the barn while the brooder end received fresh bedding creating a mixed or dual environment in the barn. Using a combination of *Salmonella* MPN quantitation, selective plating, and serotyping they detected a trend for lower levels of *Salmonella* as well as lower serovar diversity in the re-used litter when compared with the new litter environment. They associated this decline with the lower water activity of the re-used litter. When Oladeinde et al. (2025b) sampled litter from 2 commercial flocks and compared moisture levels they reported that higher levels of moisture correlated with the likelihood of a positive *Salmonella* litter sample. Based on machine learning analyses they concluded that moisture was the most significant environmental predictor of *Salmonella* presence in poultry litter. They hypothesized that the influence of moisture was related to nutrient availability.

Differences in *Salmonella* genetics may also play a role. Oladeinde et al. (2018) followed the genetic evolution of *S*. Heidelberg inoculated in poultry litter microcosms over 14 days using whole genome sequencing followed by single nucleotide polymorphism (SNPs) and InDel analysis to track genetic differences. Based on these analyses, they concluded that hotspot mutations and ColE1 plasmids acquired over time contributed to the ability of S. Heidelberg to persist in the poultry litter microcosms. When Oladeinde et al. (2025a) inoculated a cocktail of 3 *S*. Heidelberg strains consisting of antimicrobial resistance and susceptible variations into fresh pine wood shavings they noted that both plasmids and bacteriophage influenced persistence over 21 days. *Salmonella* serovars Typhimurium, Infantis, and Kentucky also appear to possess large self-conjugative plasmids encoding iron siderophore production that appear to enhance fitness in litter environments (Oladeinde et al., 2025b). More recently, Oladeinde et al. (2026) demonstrated that both plasmids and bacteriophage were determinants to whether *S*. Enteritidis contained resistance associated parallel mutations promoting stress adaptation. They concluded that bacteriophage in the poultry litter microcosm could be exerting selective pressure on *S*. Enteritidis and that phage persistence was a function of the litter bacterial community. Whether such interactions between phage and *Salmonella* in litter occurs for other *Salmonella* serovars would be of interest and may explain some of the diversity of *Salmonella* serovars observed in poultry production. This could be important from a detection standpoint since the method of *Salmonella* isolation has been identified as a factor in interpreting poultry litter results (Hoffmann et al., 2026).

The composition and metabolic activities of the indigenous litter microbial community may limit the ability of *Salmonella* to persist in older litter versus new litter. Early studies by Fanelli et al. (1970) suggested that litter microbiota or inhibitory substances in used litter could be inhibitory to *Salmonella*. This concept of microbial anti-*Salmonella* activity in litter is supported by the conclusion of Vaz et al. (2017) that recycled boiler litter subjected to shallow fermentation resulted in elimination of *S*. Enteritidis within 12 days. Older litter has also been attributed to promoting cecal microbial diversity in young chicks and in turn, promoting exclusion of *S*. Enteritidis (Ekunseitan et al., 2025). Bucher et al. (2020) expanded on this concept using microbiome RNA gene sequencing analyses to identify re-used poultry litter organisms with competitive exclusion potential and whole genome sequencing to identify candidate organisms against *S*. Heidelberg. From this study they identified a *Bacillus subtilis* isolate that exhibited increased growth when inoculated in an *in vitro* microcosm concomitant with decreases in *S*. Heidelberg. The specific relationships remain unclear between the litter microbiota and *Salmonella*. Guttierez and Schneider (2022) compared irradiated poultry litter microcosms with natural microcosms to distinguish the impact of indigenous litter microbiota versus sterile microcosms on survival of *S*. Typhimurium over 6 days at a pH of 8.0, water activity of 0.92, and temperature set at 30 ${}^{\circ }$C. They concluded that while the presence of indigenous litter microbiota under these conditions did inhibit *S.* Typhimurium survival this outcome could be different under more favorable environmental conditions. To resolve this, future studies will need to combine microbiome compositional analyses with methods such as metabolomics to delineate the functional properties that are responsible for indigenous litter microbial interactions with *Salmonella*.

## Poultry management practices that impact pathogen persistence in litter

Pathogens within the poultry house should be viewed as part of the broader litter ecosystem rather than as isolated contaminants. Their survival, persistence, proliferation, and transmission are shaped by the same environment that impact the non-pathogenic microbiota, including moisture, oxygen availability, pH, substrate availability, bird activity, among others (Wang et al., 2025; Ricke et al., 2026b). While several of these environmental conditions and poultry litter microbial responses have been extensively characterized, less is known on how poultry house management practices influence poultry litter microbial populations. These are listed in Table 2 and include attempts to limit ammonia production in poultry houses, along with impact of feed spillage, exposure to water lines, and overall bird management just prior to processing. While some of these have obvious direct impacts on litter pathogen ecology other management practices may initially be less obvious but still influence pathogen survivability and persistence.

**Table 2:** Environmental and management drivers that shift poultry litter from bedding to pathogen-permissive habitat.

**Driver/event**	**How it changes litter ecology**	**Pathogens most relevant**	**What to measure**	**Management or research implication**
Moisture accumulation	Increases water activity, microbial metabolism, manure decomposition, caking, and pathogen survival	*Salmonella, E. coli, Enterococcus, C. perfringens, Campylobacter*	Litter moisture, water activity, caking score, pH, ammonia, pathogen culture/qPCR	Treat wet/caked zones as ecological hotspots, not just bedding defects
Ammonia and pH shifts	Alters microbial survival directly, damages bird respiratory/immune function indirectly, and reflects nitrogen turnover	*Salmonella, E. coli*, respiratory/opportunistic pathogens	Ammonia ppm, litter pH, total nitrogen, uric acid/urea indicators, bird health outcomes	Link ammonia control to both welfare and pathogen susceptibility; maintain dry litter and ventilation
Feed spillage and nutrient input	Adds carbohydrates, proteins, minerals, and microbial substrates that support microbial growth	*Salmonella, E. coli*, general heterotrophs	Feeder-line litter, organic matter, nutrient chemistry, bacterial counts	Sample feeder-line zones separately from bulk litter
Water-line leaks/biofilms	Creates persistent wet bands and may seed pathogens through drinking systems	*Salmonella, Campylobacter, E. coli*	Water-line swabs, biofilm sampling, drinker-line litter, moisture, pathogen testing	Pair water sanitation data with litter microbiology
Disease or enteric disturbance	Alters fecal output, moisture, microbial shedding, and local litter chemistry	*C. perfringens, E. coli, Enterococcus, Salmonella*	Fresh feces, cecal contents, wet/caked litter, pathogen qPCR/culture, 16S sequencing	Treat disease events as ecological disturbances that may leave a microbial signature
Mortality spike	Indicates localized health breakdown, altered bird movement, and potential carcass/fecal microbial input	*E. coli, Enterococcus, Staphylococcus, Clostridium* spp.	Litter near mortality zones, dust, fecal indicators, ammonia, moisture	Sample around event locations rather than relying only on composite litter samples
Feed withdrawal, catching, crating, transport	Disturbs litter, increases floor contact, redistributes dust, and links house microbiology with processing risk	*Salmonella, Campylobacter, E. coli, Enterococcus*	Litter, feet, feathers, crop contents, crate swabs, carcass rinses, WGS/SNP tracking	Integrate preharvest litter sampling with transport and processing samples

### Managing ammonia in poultry litter: antimicrobial pressure versus bird health stress

Poultry litter is chemically composed of water, carbon, nitrogen, phosphorus, and potassium with minor quantities of chlorine, manganese, iron, magnesium, sodium, and arsenic, among other elements (Edwards and Daniel, 1992). In general, litter chemical composition is expected to influence microbial compositional fluctuations. However, nitrogen transformations have probably received the most attention. This is because one of the more critical issues related to litter and waste buildup is the accumulation of nitrogen byproducts within the bedding of a poultry house (Salim et al., 2014; Swelum et al., 2021). High crude protein diets fed to meet the nutrition, and performance demands of birds also leads to increased manure nitrogen and in turn, greater ammonia levels (Bist et al., 2023). Nitrogen in poultry manure primarily consists of uric acid and undigested proteins, which serve as substrates for microbial metabolism and ammonia production (Swelum et al., 2021). Uric acid alone accounts for approximately 50 to 70% of the total nitrogen content of poultry excreta and is the primary source of subsequent ammonia production in the litter (Nahm, 2003; Swelum et al., 2021).

The presence of ammonia can influence microbial pathogens and pathogenesis in birds both directly and indirectly. Ammonia can directly inhibit pathogens such as *Salmonella* in litter. Early work by Turnbull and Snoeyenbos (1973) demonstrated that as poultry litter ages, pH levels rise and ammonia concentrations increase, leading to salmonellacidal responses. However, they noted that different serovars varied in their susceptibility. This is consistent with the observation that adding urea to broiler litter reduced microbial contamination, with a 2% urea treatment effectively eliminating detectable *Salmonella* and *Clostridium* populations during storage (Lober et al., 1992). Himathongkham et al. (2000) demonstrated that the presence of free ammonia could inactivate *S*. Typhimurium in poultry manure as well as when added directly to peptone water or bovine manure. By comparing inhibition constants (K*i*) of *S*. Typhimurium for various ammonium salts based on batch culture growth rates, Ricke et al. (1997) determined that it was more sensitive to certain ammonium salts versus others, but initial inhibition was dependent on external pH. Williams et al. (2012) observed that decreasing sodium bisulfate amended litter pH led to decreased ammonia production and increased viability of *Salmonella* serovars. More recently, Gutierrez et al. (2022) demonstrated that urease-producing bacteria could produce NH_3_ that would inhibit *S*. Typhimurium at alkaline pH levels of more than 9, and that un-ionized ammonia was inhibitory above 0.04 M, leading them to suggest high ammonia levels in poultry litter could decrease the risk of *Salmonella* contamination of litter applied to soil.

The presence of high levels of ammonia in litter and the poultry house may also indirectly influence bacterial pathogens and poultry health (Carlile, 1984). Ammonia can significantly increase immunological vulnerability in poultry. Elevated NH_3_ concentrations have been shown to impair immune function by damaging primary lymphoid organs and impact immune responses (Wei et al., 2015; Rahman et al., 2024). Rahman et al., 2024 observed an active immune response to ammonia exposure including clustering of CD4+ and CD8+ lymphocytes along with activation of the TLR-7/MyD88/NF-κB signaling pathway and the NLRP3 inflammasome in chicken thymus tissues. Atmospheric NH_3_ not only alters bird behavior and increases mortalities but predisposes broiler chickens, laying hens, and turkeys to respiratory disease and secondary infections, including Newcastle disease, infectious bursitis, and *E. coli*, leading to damage of the respiratory tract by subsequent infectious agents (Nagaraja et al., 1984; Miles et al., 2004; David et al., 2015; Liu et al. 2020; Swelum et al., 2021). Alongside microbial concerns, atmospheric NH3 concentrations greater than 25 ppm have been shown to reduce broiler body weight by up to 6 to 9% at high concentrations, with mortality rates increasing by 8.1% (from 5.8 to 13.9%) at 75 ppm compared to controls (Miles et al., 2004). Managing ammonia concentrations at or below 25 ppm improves bird performance and reduces mortality associated with NH_3_-related health concerns and microbial susceptibility. If not accounted for, inadequate ventilation and poor litter management result in ammonia exposure, leading to tissue damage, increased susceptibility to disease, impaired flock performance, decreased carcass quality, and reduced egg quality (Weaver Jr. and Meijerhof, 1991; Swelum et al., 2021; Bist et al., 2023).

Bist et al. (2023) has categorized poultry house ammonia mitigation as either pre-excretion (namely, reduction at the source such as diet before manure is produced) and pre-release (decreasing NH_3_ production by litter amendment or removal by house design before NH3 enters into the environment). Effective pre-release control of ammonia requires proper litter management and adequate ventilation throughout the production cycle. Miles et al. (2011) concluded that maximum NH_3_ production occurred between 37.4 and 51.1% litter moisture but depended on litter temperature. Maintaining dry litter conditions limits microbial degradation of uric acid and reduces NH_4_/NH_3_ production, while sufficient ventilation removes excess NH3 from the poultry house environment (Weaver Jr. and Meijerhof, 1991; David et al., 2015; Swelum et al., 2021). Wet litter has been shown to favor *Salmonella* growth and transmission, whereas litter drying introduces osmotic and desiccation stress, reducing bacterial viability (Himathongkham and Riemann, 1999; Chen et al., 2015). Regarding air ventilation, air scrubbers have been shown to effectively remove NH_3_ from poultry house systems by passing contaminated air through an acid-based solution (Moore et al., 2018; Bist et al., 2023). More recently, indoor air scrubber systems using sulfuric acid designed specifically for poultry houses have achieved NH_3_ reductions of 87 to 99%, (Moore et al., 2025). As further technological improvements are made, it is anticipated that there is even more potential to improve in-house air quality and reduce bird and worker exposure to elevated NH3 concentrations.

In addition to developing technologies to remove NH_3_ in poultry house emissions, there has been considerable research on potential litter chemical and biological amendments that when applied reduce NH4/NH3 production (Wang et al., 2025). Chemical litter amendments including alum, organic acids, formalin, sodium bisulfate, sodium sulfate, and sulfuric acid are commonly incorporated into bedding materials have been shown to varying levels of effectiveness to mitigate NH_4_ to NH_3_ to volatilization by modifying pH (Bist et al., 2023; Obe et al., 2023; Wang et al., 2025). These chemicals control NH_4_/NH_3_ production either inhibiting microbial growth to stop uric acid degradation and preventing NH_4_/NH_3_ production or combining with and neutralizing NH_3_ (Carlile, 1984; Salim et al., 2014). Some of these chemicals elicit multiple effects on poultry litter NH_3_ production. For example, bedding materials containing zeolite–clay–rice husk ash has demonstrated the ability to reduce litter moisture, lower litter pH, and decrease NH_3_ concentrations (Nguyen et al., 2026). Broilers reared with the zeolite litter amendment exhibited reduced physiological stress, lower heterophil-to-lymphocyte ratios, lower serum cortisol levels, and fewer footpad lesions compared to those reared on conventional bedding materials due to the lower relative humidity (Nguyen et al., 2026). Alongside this, some biological-based mitigations such as feed additive egg yolk antibodies have been explored to prevent microbial uricase capacity to reduce NH_4_/NH_3_ concentrations in broiler houses (Kim et al., 2013). This potentially includes direct treatment of poultry manure with the antibody, antibody supplementation to poultry diets, or direct uricase immunization of birds (Kim et al., 2013). Park et al. (2016) demonstrated that administering *E. faecium* as a probiotic to laying hens not only increased nutrient retention and reduced nutrient excretion but also led to reduced excreta NH_3_ emission due to a shift in the fecal microbiota. Atapputu et al. (2017) compared an enzymatic biocatalyst and a bacterial culture that inhibits uricase producing organisms on broiler house NH_3_ levels and determined that enzymatic treatment was more effective. Such et al. (2023) demonstrated that a symbiotic treatment consisting of probiotics and prebiotics fed to broilers resulted in decreased uric acid content and the urinary N ratio of the excreta.

### Potential contribution of feed spillage to litter microbial ecology

The presence of poultry feed in the poultry house represents another potential factor that can have both direct and indirect influences on litter pathogen dissemination and microbial ecology. Indirectly, feed can impact the litter microbial ecology by modulating the GIT microbial composition of birds during the production cycle (Olson et al., 2022a). This is in part due to poultry feeds possessing characteristic microbial ecologies, which may contribute to the development and maturation of a chicken's GIT microbiota (Olson et al., 2022a). Feed ingredients harbor diverse microbial communities, some of which overlap substantially with those found in the ileum, cecum, and excreta of broiler chickens (Haberecht et al., 2020; Olson et al., 2022a). This is not too surprising, since litter-based microorganisms can also populate the GIT of young birds once they are exposed to the litter (Wang et al., 2016).

Of the pathogens, *Salmonella* specifically is a prevalent source of feed contamination, able to persist within feed storage for weeks and spread within production feed facilities and can lead to *Salmonella* colonization of the poultry GIT (Ha et al., 1998; Maciorowski et al., 2004; Jones, 2011). In addition, contamination of feed may originate from numerous points throughout production, including transportation, storage facilities, and other environmental sources encountered during manufacturing and handling (Olson et al., 2023; Ricke et al., 2026c). Feed contamination is one of several sources of *Salmonella* exposure within flocks (Maciorowski et al., 2004; Obe et al., 2023). It has been shown that poultry feed artificially contaminated with *S.* Typhimurium could infect birds consuming the feed (Yang et al., 2017), and thus, it is quite possible that a continuous loop of feed-bird-litter contamination follows the inoculation of newly introduced birds. Efforts to limit *Salmonella* contamination in feeds are warranted, but the risk of recontamination during the later stages of feed processing remains (Maciorowski et al., 2004; Jones, 2011).

Locatelli et al. (2017) determined the spatial variability of the poultry litter and fecal microbiomes from distinct areas within a poultry house by sampling near cooling pads, waterer/feeder lines, bulk litter, and near fans. They concluded that for fecal and litter microbiomes genus-level Operational Taxonomic Units relative abundances were significantly different based on sampling area within the house. Winkler et al. (2017) compared broiler house litter samples from around the feed line, water line, and the north wall of the poultry houses. They detected the lowest levels of total coliforms and *E. coli*, near the feed line versus the water line, but enterococci and *C. perfringens* were intermediate between the water line and the wall. The litter around the feed lines contained the least amount of moisture of the 3 sites sampled. This would suggest that the microbial ecology of the litter is not equally distributed throughout the house and that there may be areas such as feeders and waterlines where different microbial populations are enriched. More recently, the contribution of feed to local litter microbial ecology has been proposed to influence microbial gradients in nutrient-rich zones surrounding feeders within pens (Peckenschneider et al., 2026). Tasistro et al. (2004b) reported considerable variation in mineral content of litter near feeders in commercial houses. In addition, Tasistro et al. (2004a) observed the lowest amount of litter decomposition near the feeders in a commercial house. Consequently, feed could be a source of microbial contaminants to litter and indirectly impact litter microbial ecology by altering the litter's chemical composition. As feed is continuously distributed and spilled throughout production, it would potentially provide a constant available nutritional source that supports localized microbial growth and survival in the litter areas that are in proximity to the feeder. By introducing concentrated carbohydrates, proteins, and minerals directly into the bedding material, spilled feed could provide ideal substrates to support the proliferation of both nonpathogenic and pathogenic microorganisms. However, to characterize the impact of feed spillage on litter microbial ecology will require biomapping of the litter floor of the poultry house with a combination of chemical and microbial analyses. Peckenschneider et al. (2026) developed a grid system to construct three-dimensional plots of small layer hen pens for biomapping the litter in the pens. Using a grid system allowed them to theoretically plot a variety of inputs including microbial counts, pH, moisture, and litter chemical compositional profiles and statistically analyzed to determine relatedness with different regions of the pen such as feeders, water lines, and bird enrichment areas. However, this represents a small-scale attempt at plotting the distribution of variables impacting litter and developing a large-scale version for commercial poultry houses is a potential sampling challenge.

### Water lines and biofilm formation

Water lines in poultry houses may also be a potential local reservoir for pathogens and nonpathogens in poultry house litter. This may in part be due to their ability to form biofilms. For example, *Salmonella* strains with high biofilm productivity demonstrate substantially greater long-term survival during desiccation than biofilm-deficient strains, in part due to the protective effects of extracellular polymeric matrices (Steenackers et al., 2012; Coughlan et al., 2016). In addition, the ability to potentially form biofilms enables organisms including *Salmonella* to persist in environments and resist disinfectants (Steenackers et al., 2012; Coughlan et al., 2016). *Salmonella* has been detected within water line biofilms, where attachment to surface-associated microbial communities provided protection from environmental stressors and sanitation procedures (Ogundipe et al., 2025). As cells are periodically released into the water supply, these pathogens can be present as a potential source of horizontal transmission and dissemination within poultry houses, providing constant bacterial exposure to flocks if not accounted for by poultry house hygienic practices. Under controlled experimental conditions Yang et al. (2017) demonstrated that drinking water inoculated with *S*. Enteritidis colonized the ceca of young broiler chicks.

Only a few studies have attempted to examine water lines and litter in commercial poultry houses. Ogundipe et al. (2025) compared the sequenced microbiome 16S rDNA communities of commercial poultry house litter environment and water line biofilms from 2 groups of poultry farms. These commercial farms consisted of 5 farms with normal sulfur-iron water quality and 5 with a history of high sulfur-iron water quality. They also quantified and identified *Salmonella* serovars from the 2 sets of environments. Overall, they observed a much higher incidence of *Salmonella* in the litter samples (96.7% incidence rate of *Salmonella* and an average of 4.1 Log10 CFU/sample than the water line biofilm (4.2%, incidence rate and an average of 0.5 Log10 CFU/sample) with Kentucky, Enteritidis, Typhimurium, and Infantis being the most frequently isolated serovars. When respective microbiome profiles were compared, they detected distinct differences microbial community compositional profiles in the litter with *Aerococcus, Lactobacillus*, and *Staphylococcus* being dominant. However, for water line biofilms the normal sulfur-iron water quality biofilms contained primarily probiotic *Bacillus* whereas pathogenic *Bacillus* occurred in water line biofilms from farms using high sulfur-iron water quality.

Once established, biofilms are often difficult to eradicate through conventional cleaning and disinfection procedures due to the protective extracellular polymeric matrix surrounding embedded cells (Galié et al., 2018). Ogundipe and Obe (2025) compared peracid-based, peroxide-based, and hypochlorite-based sanitizers against field-isolated *Salmonella, E. coli,* and *Bacillus* on their respective antibiofilm efficacies on polyvinylchloride pipe. These bacterial biofilms were evaluated for each bacterial strain individually as well as part of a mixed culture. They observed that both peracid and hypochlorite-based disinfectants eliminated the biofilms of all strains tested at the minimum inhibitory concentration in mixed-species cultures while peroxide-based disinfectant did not elicit a significant effect. Overall, they concluded that peracid had the greatest potential as a drinking water sanitizer, due to its efficacy against both planktonic and biofilm bacterial cells. Alternative disinfectants that are considered natural compounds such as essential oils and phenolic compounds have also been suggested for reducing *Salmonella* biofilms in poultry production (Nesa et al., 2025).

While there were differences in the litter compared to water line microbial composition observed by Ogundipe et al. (2025) further studies are needed to refine the sampling strategy. For example, Winkler et al. (2017) sampled different locations within a commercial broiler house and concluded that total coliforms, *E. coli*, and *C. perfringens* were positively correlated with litter moisture content and were higher around the water lines. Tasistro et al. (2004a) reported that litter samples taken near waterers showed evidence of very advanced decomposition under anaerobic conditions. In a later study, Miles et al. (2013) found that poultry litter close to waterers emitted the most cumulative NH_3_ with less NH_3_ emitted from litter near feeders and sidewalls. Future research based on more precise localized locations in the poultry house such as a grid system as described by Peckenschneider et al. (2026) could provide more detailed information on litter ecology as a function of location and gradient. This coupled with whole genome sequencing to compare organisms from the water lines and the litter may reveal how extensively litter and waterers become cross contaminated.

### Poultry flock management impacts

Certain poultry farm management practices can influence the poultry house environment and can lead to proliferation of pathogens such as *Salmonella* (Obe et al., 2023; Neelawala et al., 2024). Practices that allow mortality and disease outbreaks represent severe biological disturbances within the poultry litter system because they signal a breakdown in flock health and a potential reorganization of the litter floor. These events are associated with increased pathogen shedding, altered bird movement and behavior, changes in fecal output, and, in some cases, carcass-associated microbial inputs. Rather than functioning as isolated health incidents, mortality and disease episodes should be viewed as high-risk ecological windows during which the stability of the litter’s microbial and chemical profile may be disrupted, creating conditions that favor the recovery or amplification of opportunistic taxa. The poultry house environment, including litter, feces, and dust, can serve as a reservoir for a wide range of organisms including pathogens (Terzich et al., 2000; Lu et al., 2003; Dumas et al., 2011; Blżażejewska et al., 2022). During disease events, fecal material from compromised birds may increase the local abundance of these organisms, particularly in areas where moisture and manure deposition already support microbial activity. For example, *C. perfringens* is associated with nutrient-rich, oxygen-limited, and disease-associated conditions, where it can proliferate and increase the risk of necrotic enteritis (Van Immerseel et al., 2004; Moore, 2016; Broom, 2017; Fathima et al., 2022). In built-up litter systems, these risks may extend beyond a single flock because heat-tolerant or spore-forming organisms can persist through downtime and contribute to recolonization of the house floor. However, few studies have directly paired the timing of mortality events with high-resolution litter microbiome, pathogen, and environmental data. Therefore, the idea that mortality-associated disturbances amplify opportunistic taxa remains an important hypothesis and a key research gap, as much of the current evidence reports pathogen prevalence without resolving the timing, location, and ecological conditions that drive these microbial shifts. The final stages of the production cycle, particularly feed withdrawal, catching, transport, and crating, represent critical late-flock disturbances that could connect litter ecology with food safety. These events should not be considered only as logistical steps before processing; they are biological and physical disruptions that simultaneously alter bird physiology, bird behavior, litter contact, and microbial transfer. During this period, increased movement, handling stress, altered fecal and cecal output, and intensified contact with the floor can increase the likelihood that microorganisms will move from litter to birds and, eventually, into the processing environment.

Feed withdrawal is a standard preharvest practice that can strongly influence the bidirectional microbial interaction between the bird and the floor (Rasekh et al., 2005). As birds stop feeding, the GIT undergoes physiological changes that may affect pathogen colonization, persistence, and shedding (Burkholder et al., 2008). Several studies have reported increased recovery of *Campylobacter* and *Salmonella* from the crops of feed-withdrawn broilers, indicating that feed withdrawal can alter pathogen presence in the upper GIT (Ramirez et al., 1997; Byrd et al., 1998; Corrier et al., 1999a,b). Buhr et al. (2017) further showed that both the litter environment and the broiler’s *Salmonella* status prior to feed withdrawal can contribute to crop *Salmonella* recovery, supporting the idea that litter can serve as a potential source of ingested pathogens during the preharvest period. These findings reinforce the role of the poultry house floor as a preharvest microbial reservoir, where even a brief feed-withdrawal window can create measurable consequences for microbial exchange between litter and the GIT of the bird. This risk may be further influenced by transportation duration following feed withdrawal, which may impact overall carcass bacterial loads.

Catching and crating add a major mechanical disturbance to this late-flock risk window. The physical handling of birds disturbs the litter pack, aerosolizes dust and microorganisms, redistributes caked and friable material, and potentially increases contact between contaminated floor material and bird feathers, feet, skin, and crates. Transport crates have been identified as a source of *Salmonella* and *Campylobacter* cross contamination as broilers are moved from the farm to the processing plant (Slader et al., 2002; Reina et al., 2024) This indirectly supports the idea that late-flock handling can transfer microorganisms from the house environment into the broader production chain. In this way, the microbial history of litter could be physically carried out of the house and into transport and processing environments. The farm-to-processing connection is supported by studies such as Berghaus et al. (2013), who measured *Salmonella* and *Campylobacter* in both environmental farm samples and processing plant carcass rinses. They concluded that litter had a strong association between *Salmonella* loads in pre- and postchill carcass rinses. Together, these findings indicate that when high pathogen burdens in litter coincide with feed withdrawal, catching, transport, and crating, poultry litter could become an important contributor to downstream food safety risk.

The purpose of characterizing these late-flock events is not to suggest that litter is the sole source of carcass contamination, but rather to recognize it as a critical potential preharvest reservoir that interacts with bird physiology, handling, and transport. However, future studies need to focus not only on general longitudinal dissemination patterns of *Salmonella* and other pathogens in poultry production but employ methods that allow better resolution of individual serovars and strains. Certainly, advanced molecular methods such as whole genome sequencing offer the ability to differentiate pathogen strains. Whole-genome sequencing or SNP-based phylogenetic analysis of *Salmonella* isolates recovered across farm, transport, and processing environments, as well as high-resolution *Salmonella* approaches such as CRISPR-SeroSeq have been used to identify *Salmonella* strains in poultry flocks (Siceloff et al., 2022; Quinn et al., 2023; Adhikari et al., 2025). Genomic barcoding has also been used in controlled *Salmonella* challenge studies to track multiple *Salmonella* strains simultaneously (Yang et al., 2018). Collectively these studies have revealed that *Salmonella* serovars and strains can change during a poultry production cycle potentially and could be due to differences in environmental fitness and/or competitive exclusion of one strain by another strain. The key question is determining whether changes in litter during poultry production impact the diversity of *Salmonella* serovars and strains. This includes poultry production management practices such as incorporating different types of litter amendments, age of the litter, and the litter environment during feed withdrawal.

## Conclusions

Poultry litter is a continually evolving ecosystem that functions as both a recipient and source of microorganisms within poultry houses (Fig. 1). As bedding accumulates excreta, moisture, feed residues, and other organic materials, conditions favor the persistence and transmission of bacterial pathogens alongside the accumulation of ammonia. Pathogenic organisms, including *Clostridium,* pathogenic *Escherichia coli, Staphylococcus, Campylobacter, Enterococcus,* and *Salmonella*, can survive within litter and contribute to disease transmission. Poultry flock management practices can impact environmental conditions such as litter moisture, temperature, ventilation, and re-use practices that subsequently influence microbial survival and flock exposure. Ultimately, effective poultry production management should consider litter management practices to limit pathogen persistence and promote conditions unfavorable to their dissemination to prioritize poultry health as well as reduce public health concerns.

## Funding information

This work was supported by AgriGro. The funder had no role in the writing of the manuscript or in the decision to submit the review for publication.

## Author contributions

S.C.R. drafted the manuscript. E.G.O. created the revised Fig. 1. All authors (A.E.R., E.G.O., and B.M.H.) contributed to manuscript revision, reviewed the final version, and approved the manuscript for submission.

## Data availability

No new data were generated or analyzed in this review.

## Disclosures

The authors S.C.R. and E.G.O. declare that they have no known competing financial interests or personal relationships that could have appeared to influence the work reported in this manuscript. Coauthor B.M.H. is employed by AgriGro, Inc., Doniphan, MO, 63935, USA but declares no competing interests.
